# Occurrence and Exposure Assessment of Rare Earth Elements in Zhejiang Province, China

**DOI:** 10.3390/foods14111963

**Published:** 2025-05-30

**Authors:** Shufeng Ye, Ronghua Zhang, Pinggu Wu, Dong Zhao, Jiang Chen, Xiaodong Pan, Jikai Wang, Hexiang Zhang, Xiaojuan Qi, Qin Weng, Zijie Lu, Biao Zhou

**Affiliations:** 1 School of Public Health, Ningbo University, Ningbo 315211, China; 17867970961@163.com; 2 Zhejiang Provincial Center for Disease Control and Prevention, Hangzhou 310051, China; rhzhang@cdc.zj.cn (R.Z.); pgwu@cdc.zj.cn (P.W.); dzhao@cdc.zj.cn (D.Z.); jchen@cdc.zj.cn (J.C.); xdpan@cdc.zj.cn (X.P.); jkwang@cdc.zj.cn (J.W.); hxzhang@cdc.zj.cn (H.Z.); xjqi@cdc.zj.cn (X.Q.); 881012022119@hmc.edu.cn (Q.W.); 130232023409@hmc.edu.cn (Z.L.); 3 School of Public Health, Hangzhou Medical College, Hangzhou 310013, China

**Keywords:** rare earth elements, pollution, dietary exposure, risk assessment

## Abstract

In this study, we aimed to investigate the occurrence of rare earth elements (REEs) in commonly consumed foods and assess the dietary exposure risks among different age groups in Zhejiang Province. The results showed that tea and shrimp had the highest REE detection rates, reaching 100%. Of all the food categories examined, tea exhibited the highest REE concentrations, significantly exceeding those in other foods. This may be attributed to differences in moisture content, root absorption mechanisms, and processing methods. The concentration pattern of REEs in all samples occurred in the following order: cerium > lanthanum > yttrium > neodymium > neodymium > scandium > praseodymium > gadolinium > dysprosium. The light REEs/heavy REEs (HREEs) ratio was consistently > 2 but remained lower than the ratios observed in the soil and sediments, indicating a potential risk of HREE enrichment. Dietary exposure assessments revealed that the total REE intake among Zhejiang residents was below the established safety threshold (51.3 µg/kg BW/day), with children experiencing the highest exposure (3.71 µg/kg BW/day), primarily due to their lower body weight. In the assessment of individual rare earth elements, Ce exposure in children aged ≤ 6 years exceeded the toxicological reference value. However, this threshold was established based on studies in pregnant and lactating populations and might not be directly applicable to young children. Therefore, overall dietary exposure to individual REEs remains within safe limits. REE exposure from tea consumption did not pose a health risk, even for habitual tea drinkers. These findings underscore the importance of continuous monitoring of REE accumulation in food and additional research on the potential long-term health effects, even though the current exposure levels of REEs are below the established safety limit. This is especially important considering the bioaccumulative nature of REEs and the limited paucity of toxicological data, particularly in vulnerable populations.

## 1. Introduction

Rare earth elements (REEs) are a group of lanthanides with atomic numbers ranging from 57 to 71, along with scandium (Sc) and yttrium (Y) [1], which naturally coexist in mineral deposits. Based on their atomic structures and physicochemical properties, REEs are categorized into light REEs (LREEs) and heavy REES (HREEs). These elements are widely used in multiple industries due to their excellent magnetic, optical, and electrical properties [2], essential for improving product performance and production efficiency. In addition, REEs are used in specific agricultural applications, such as in certain fertilizers and feed additives, possibly as trace elements or to promote specific growth characteristics [3,4]. Global REE production has surged since the 1950s, and the demand is projected to increase by 2600% over the next 25 years [5]. As of 2024, China accounts for the largest share of the global REE supply, at 49% (44 million tons), with an annual production of 270 thousand tons [6].

The increasing release of REEs into the environment due to their development and application has led to concentrations in various environmental media that may be several orders of magnitude higher than background levels [7]. REE contamination has been found in the soil and river sediments of many mining areas, such as the Ganzhou mining area [8], Amazon gold mining area [9], Xiangjiang River [10], Yellow River [11], and Santos Estuary (Brazil) [12]. REEs enter the environment through activities such as mining and fertilization, significantly elevating their levels in the soil, water, and air [7,13]. In soil, REEs are absorbed by crops, and they accumulate in the edible parts [14], whereas in aquatic environments, they bioaccumulate in organisms, such as fish and shellfish, through water and sediment exposure [15]. These findings underscore the potential risks of REEs entering the food chain and the need for ongoing monitoring and management [16,17].

Oral intake is a major route through which REEs enter the human body. After ingestion, REEs pass through the digestive tract and enter the bloodstream, where they are distributed to organs, such as the liver, kidneys, and heart, as well as the nervous and reproductive systems [18]. REEs are mainly absorbed in their ionic form or as complexes with organic molecules such as amino acids and proteins [19]. REEs can interact with cell membranes within these organs and affect cellular functions, leading to negative effects such as neurotoxicity, pulmonary toxicity, nephrotoxicity, and cytotoxicity [20,21,22,23]. Animal studies have demonstrated that high concentrations of REEs can damage the nervous system, lungs, kidneys, and cellular structures. In addition, several studies have suggested that maternal exposure to REEs may be associated with an increased risk of premature membrane rupture [24], and early exposure to REEs may increase the risk of gestational diabetes [25]. These findings emphasize the need for further research on the relationship between REE exposure and maternal health risks, especially through long-term epidemiological data, animal models, and multicenter collaborations, to delve deeper into this issue.

In China, REEs have been widely detected in various food products, including tea [26], vegetables [27], fruits [28,29], aquatic products [15], and grains [30]. For example, the average REE concentration in Chinese tea varies across regions, with Henan Province’s black tea at 2.960 mg/kg, Shanxi Province’s green tea at 2.500 mg/kg, Guizhou Province’s oolong tea at 3.870 mg/kg, and Hunan Province’s black tea at 2.955 mg/kg [26]. Similarly, the REE content of vegetables and fruits differed between mining and non-mining areas [28]. The average REE concentration in vegetables in the mining and non-mining areas was 92.90 μg/kg and 62.38 μg/kg, respectively. The REE content in fruits from mining areas was 12.90 μg/kg, compared to 11.89 μg/kg in fruits from non-mining areas. Aquatic products, such as mantis shrimp from Shandong Province [31], have an average REE content of 9.60 μg/kg. Grains also reflect significant variations, with rice from Jiangxi mining areas containing 102.79 μg/kg of REEs, while rice from non-mining areas contains only 35.28 μg/kg [32]. Furthermore, other studies have also investigated the REE levels in food samples from specific regions. For example, the concentration of ΣREE in edible mushrooms in Poland was 0.04 mg/kg [33], the average level of ΣREE in samples from terrestrial (plant feed [containing unprocessed materials like barley, wheat, oats, bran, and fodder], fruits, honey, wild animal liver) sources in Italy was 0.46 mg/kg, and the average level of ΣREE in aquatic (seaweeds, zooplankton, bivalves, fishes) sources was 3.12 mg/kg [34]. There are fewer studies on REEs in food products from abroad, and this may be due to the distribution of rare earth resources. These findings highlight the overall impact of mining activities on REE contamination levels [28], with certain foods, such as tea, leafy vegetables, and aquatic products, showing notably higher REE accumulation and contributing significantly to dietary exposure.

Due to the high detection rate and concentration of REEs in certain foods, dietary exposure assessment for specific foods has been conducted in some regions. Yang [35] assessed the REE concentrations in 11 food items in the Chinese diet, showing that the average daily exposure to REEs from food was 1.62 μg/kg BW, accounting for 3.14% of the recommended intake, indicating a low health risk. Song [36] used a simple distribution model (deterministic assessment) to calculate the daily exposure to REEs per kilogram of body weight for individuals. The results indicated that the average daily exposure from tea in the Borderlands was 0.668 μg/kg BW, which is within the safe range. One study [37] employed Monte Carlo simulations to assess the probabilistic exposure to REEs in foods from a large mining area in Shandong Province. The study’s findings showed that the daily intake of REEs ranged from 3.80 to 5.40 μg/kg BW, indicating a low level with no health risks. Overall, the results of the dietary exposure assessments suggest that REE dietary exposure risk for the general population is safe. However, due to limited toxicological data, most studies have focused on assessing total REEs, with relatively few evaluations of individual REEs, particularly heavy REEs. Strengthening single-element assessments is essential for a more precise understanding of the potential health risks. In addition, dietary exposure assessments of REEs in the Zhejiang Province remain limited, highlighting the need for further research in this region to ensure food safety and protect public health.

Zhejiang is a coastal province in eastern China with a well-developed economy, strong industrial base, extensive applications of new energy, and rare earth resource exploitation. It is a major tea-producing region; tea plays a significant role in the local culture, and its consumption is widespread. In addition, using rare earth fertilizers in agriculture, particularly in southern China, increases the risk of REE accumulation in crops, such as vegetables. Furthermore, aquaculture activities near industrial zones facilitate the transfer of REEs from water sediments to aquatic organisms. These combined factors increase the risk of REE contamination in food products such as tea, vegetables, fruits, yellow wine, and shrimp, which are commonly consumed by Zhejiang residents, highlighting the need for continuous monitoring and risk management.

Given the increasing global use of REEs and the insufficient data on human dietary intake of these elements, particularly in Zhejiang Province, we aimed to monitor REE contamination levels in five major food categories—tea, vegetables, fruits, yellow wine, and shrimp—and assess their dietary exposure risk. In previous studies, the assessment of single REEs in food was insufficient because of insufficient toxicological information. In this study, based on the existing toxicological limits of animal experiments, we conducted a dietary assessment of five REEs in five food products after conversion by uncertainty factors to obtain more accurate results of exposure to REEs and scientifically assess the potential effects of REEs on the health of the population.

## 2. Materials and Methods

### 2.1. Sample Collection

Food sampling followed the formula below [38]:

(1) $$N=\frac{Z^{2}\times \left[P\times \left(1-P\right)\right]}{d^{2}}$$

where *N* is the sample size; *Z* = 1.96 (level of confidence, 95%); *P* = 0.5 (expected contamination rate); and e = 10% (margin of error). Using this formula, we estimated that the minimum required sample size required was 96.04. Due to the lower concentration of *P* in REEs compared with other metals, the minimum sample size was expanded to 547. We collected 547 samples using a multistage stratified random sampling method from various cities in Zhejiang Province from 2018–2019, containing 20 food items from five categories (tea, vegetables, fruits, yellow wine, shrimp, and its products) (Table 1). Samples were packaged in bulk (including self-simplified packages) and pre-packaged foods and sampling places were categorized into retail (supermarkets, convenience stores, specialty stores, farmers’ markets, street vendors) and farms. Samples were sent to the laboratory as soon as possible after sampling completion, sealed, and frozen until analysis.

### 2.2. Method

#### 2.2.1. Determination of REEs Content

All laboratories followed standardized procedures for analysis, and the analytical and data entry personnel were trained before the survey. Detection of REEs in food was carried out in accordance with the National Risk Monitoring Workbook for Food Contamination and Harmful Factors and the Inductively Coupled Plasma Mass Spectrometry (ICP-MS) [39]. In accordance with the Recommended Procedure for the Credible Evaluation of Low-Level Contaminants in Foods issued by the World Health Organization [11], in the exposure assessment, non-detections were replaced with a 1/2 Limit of Detection.

The detection limits of REEs in vegetables, fruits, wine, shrimps, and their products are as follows (unit: mg/kg): Sc (0.001), Y (0.001), La (0.001), Ce (0.001), Pr (0.0005), Nd (0.0005), Sm (0.0005), Eu (0.0002), Gd (0.0003), Tb (0.0002), Dy (0.0002), Ho (0.0001), Er (0.0002), Tm (0.0001), Yb (0.0002), Lu (0.0001).

The detection limits of REEs in tea are as follows (unit: mg/kg): Sc (0.002), Y (0.002), La (0.003), Ce (0.002), Pr (0.002), Nd (0.002), Sm (0.002), Eu (0.0006), Gd (0.0007), Tb (0.0005), Dy (0.0005), Ho (0.0003), Er (0.0005), Tm (0.0003), Yb (0.0005), Lu (0.0003).

#### 2.2.2. Resident Consumption Survey

In this study, we obtained the consumption data from the 2015–2017 survey on the dietary consumption status of Zhejiang Province residents. The survey sites covered 10 counties (cities and districts) in Zhejiang Province, and the survey respondents were permanent residents who have lived there for ≥6 months. Multistage stratified random sampling was used to select the survey respondents on a household basis, and all the permanent residents of the sampled households selected to be the respondents of the survey signed an informed consent form (guardians signed on behalf of their minors), with a total population of about 10,000 respondents. The 3-day, 24 h method [40] was used to investigate the consumption of various types of food by different age groups in the past year.

#### 2.2.3. Quality Control

When determining the concentration of pollutants, sample testing must be performed in strict accordance with the national standard operating procedures. Intra-laboratory and inter-laboratory quality control was performed, and standard substances were used to examine the status of the instrument before, during, and after sample testing. For instruments without suitable interference elimination modes, interference equations were used to correct the results, and two blank experiments were performed at the same time as the measurement of each sample to ensure that the test data were accurate and reliable.

#### 2.2.4. Exposure Assessment of Consumers

Tea was assessed separately due to the need to account for REE leaching during infusion.

Tea consumer.

The risk of exposure to REEs for tea consumers was calculated using the formula:

(2) $$EDI=\frac{\sum \left(\left[REEs\right]\times IR\times LR\right)}{BW\times 1000}$$

Here, EDI is the daily intake of REEs in tea per kilogram of body weight (mg/kg BW), $\left[REEs\right]$ indicates the mean total REE concentration in a given category of tea (mg/kg), IR indicates average daily intake of tea (g/day), and LR indicates Leaching Rate of tea (%). Based on consumption survey data from Zhejiang Province, the estimated average body weights per individual were as follows: 20.0 kg (≤6 years), 33.7 kg (7–12 years), 52.4 kg (13–17 years), 62.0 kg (18–59 years), and 60.4 kg (≥60 years).

**Table 1:** Rare earth element leaching rate of 6 kinds of tea.

Tea Category	Leaching Ratio (%)	Extraction Procedure	Reference
flower herb teas	29.80	2 g tea + 200 mL boiling deionized water, infused for 30 min	[41]
oolong tea	17.4	5 g tea + 550 mL boiling deionized water for 20 min, infused 5 times	[42]
green tea	13.89	3 g tea + 150 mL boiling water for 5 min, infused 3 times	[43]
white tea	15.26	0.5 g + 250 mL 90 °C deionized water for 180 min, infused 3 times	[44]
Pu’er tea	13.59	3 g tea + 150 mL boiling water for 5 min, infused 3 times	[43]

Other food consumer.

The risk of REE exposure for other food consumers was calculated using this formula:

(3) $$EDI=\frac{REE\times CR}{BW\times 1000}$$

Here, EDI is the daily intake of REEs in vegetables per kilogram of body weight (mg/kg BW), which is the concentration of REEs in vegetables, fruits, wines, shrimp, and other products (mg/kg) calculated based on the wet weight; Cv is the concentration of total REE in each food; CR is the average daily consumption (g/day); and BW is the body weight (kg) of each individual.

#### 2.2.5. Health Risk Assessment of REEs

To assess the cumulative health risk associated with dietary exposure to total REEs, we calculated the Hazard Index (HI) using the following formula:

(4) $$HI=\sum_{i=1}^{n}{HQ}_{i}=\sum_{i=1}^{n}\frac{{EDI}_{i}}{RfD}$$

Here, HI is the hazard index, ${HQ}_{i}$ is the hazard quotient of an individual rare earth element from each food category. ${EDI}_{i}$ is the daily intake of an individual rare earth element from each food category. (mg/kg BW/day), and RfD is the reference dose.

Based on the recommendation from the Chinese Scientific Committee on Food Safety Risk Assessment [28], we adopted a conservative ADI of 51.3 μg/kg BW/day, which is the lowest value among the three major REEs (La, Ce, Y). Although this value was derived from individual elements, it was conservatively applied to total REEs exposure for preliminary cumulative risk evaluation.

If HI > 1, the potential hazard of REEs for the food is considered with caution, while HI < 1 indicates that the health risk of REEs for the food is in the acceptable range.

#### 2.2.6. Calculation of the Reference Dose for Health Risk Assessment of Individual REEs

In the absence of established reference values for most individual REEs, reference doses (*RfDs*) for La, Ce, Y, Nd, and Sm were estimated based on available sub-chronic or chronic animal toxicity data [45,46,47,48,49,50], following a previously reported the method [51].

RfDs were calculated as

(5) $$RfD=\frac{NOAEL}{UF}$$

where RfD is the reference dose, NOAEL is the no-observed-adverse-effect level obtained from animal studies; and UF is the uncertainty factor, which, under conservative considerations, has a value of 200. To account for interspecies differences, dose extrapolation, and human variability, especially in sensitive populations such as children, a conservative UF of 200 was applied [51].

*RfDs* were introduced to evaluate whether the estimated daily intake (EDI) of individual REEs exceeded toxicity-based thresholds.

### 2.3. Statistical Analysis

Data were entered and processed using Excel 2010, and statistical analyses were performed using R 4.3.3, with differences considered statistically significant at *p* < 0.05. The data on the content and consumption of REEs in various food types were skewed (*p* < 0.05), so percentiles such as P50 and P95 were used to characterize their differences in the exposure to REEs among various food types and subgroups.

In the dietary exposure assessment, considering the extreme values of consumption data obtained from the questionnaire survey, P95 was used to represent the consumption of the high consumption group, while the REE content was derived from mass spectrometry analysis, which was relatively accurate, and the maximum value was used to represent the high contamination scenario. Therefore, the REE intake in four groups of different consumption scenarios was obtained (Scenario 1—consumption P95 multiplied by REEs pollution level P95; Scenario 2—consumption P50 multiplied by REEs pollution level P95) and the intake of REEs was calculated separately for the populations of different age groups. REE intake was calculated for different age groups (≤6, 7–12, 13–17, 18–59, and ≥60 years old).

## 3. Results

### 3.1. REE Prevalence in Zhejiang Province

#### 3.1.1. REE Detection in Five Food Categories

As shown in Table 2, REEs were commonly detected in various foods, with black tea, green tea, Tieguanyin tea, and shrimp having the highest detection rates. The detection rates of LREEs and HREEs were 100%. Berries and typical tropical and subtropical fruits were also detected at a higher rate, with the detection rate of LREEs at 100% and HREEs at >90%. Except for aquatic vegetables, which had the lowest detection rate of REEs (47%), the remaining food had detection rates of >80%.

For individual element levels, lanthanum (La), cerium (Ce), and yttrium (Y) had the highest detection rates in all food types, with Ce > La > Y. Neodymium (Nd), Sc, praseodymium (Pr), gadolinium (Gd), and dysprosium (Dy) had the highest detection rates in most food products, with Nd > Sc > Pr > Gd > Dy. The remaining elements had low detection rates in most food products.

#### 3.1.2. REE Concentration in Five Food Categories

By observing Table 3 and performing Kruskal–Wallis and Dunn’s tests, we found that tea had the highest (*p* < 0.05) total REE content among the different food items, with Tieguanyin tea having a much higher (*p* < 0.05) REE content than black tea and green tea. Next, shrimp, vegetables, and fruits had higher REE contents, and yellow wine had the lowest REE content. Notably, among vegetables, young stems, leaves, and cauliflowers had much higher REE contents than other vegetables. Regarding REE enrichment, all foods showed LREE enrichment (LREEs/HREEs > 1), especially vegetables and fruits with more obvious REE enrichment (LREEs/HREEs > 3.5), followed by tea and yellow wine.

Regarding the content of individual elements from Table 3 and Table S1, in tea and shrimp, LREEs were higher for Ce, La, and Nd, of which Ce > La > Nd > Pr, and HREEs were higher for Y, Sc, Gd, and Dy, of which Y > Sc > Gd > Dy. In vegetables, fruits, and yellow wine, the LREEs show the same distribution as in tea, and the content of HREEs is almost zero.

#### 3.1.3. REE Distribution Pattern in Five Food Categories

**Figure 1:**
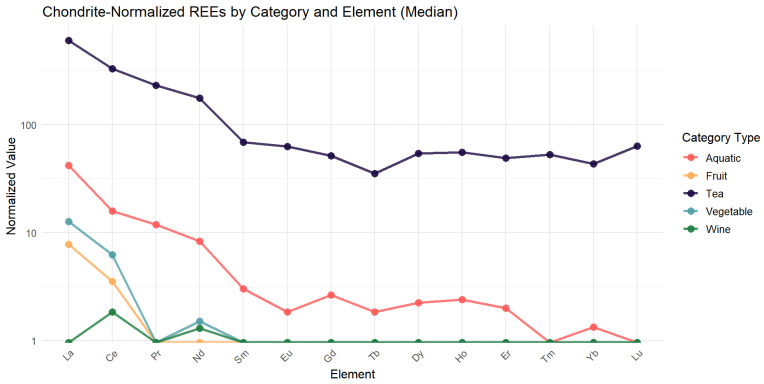
The distribution patterns of 16 REEs in different food categories.

In this study, we adopted the normalized values of chondrite [52] as a normalized reference for defining the normalized REE pattern. Figure 1 demonstrates that the overall values of the normalized REE curves for tea were high, especially in the LREEs (such as La, Ce, and Pr). Next, aquatic products have a similar distribution of normalization as tea but are lower than tea overall. Vegetables and fruits have similar normalization curves. In contrast, alcohol has the lowest normalized value and shows a negative anomaly for La.

**Figure 2:**
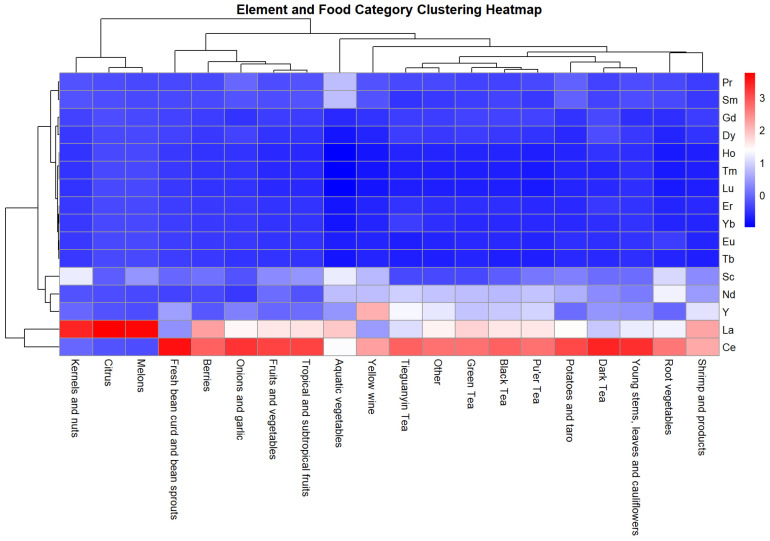
Clustering heatmap of 16 REEs in different food categories.

Figure 2 revealed distinct clustering patterns among REEs. LREEs, such as La and Ce, exhibited significantly higher concentrations (indicated by red and pink hues) compared to HREEs, including ytterbium (Yb), lutetium (Lu), and holmium (Ho), which are mostly shown in blue. Ce and La stand out as the most abundant elements across food categories, while HREEs like thulium (Tm) and Lu show minimal accumulation.

From the food perspective, REE concentrations were significantly higher in tea types (Tieguanyin, Green, Black, and Dark teas), with Tieguanyin tea showing the most intense signals, particularly for Ce and La. In contrast, categories such as root vegetables, fruits and vegetables, kernels and nuts, and fresh bean curd and bean sprouts exhibit consistently low REE concentrations, as represented by the predominance of blue shades.

### 3.2. Daily Consumption of REE Exposure Among Consumers

The Zhejiang Provincial Nutrition and Health Survey provided consumption data, as described in the Methods section. In accordance with Table 4, the food consumption of Zhejiang Province residents shows significant age characteristics. Tea consumption is dominated by Tieguanyin and Green teas, which are mainly concentrated in the adult and elderly groups, while the consumption of teenagers and children is almost zero. Vegetable consumption increases gradually with age and peaks in the older age group. Fruit consumption remained stable across age groups, with a slight increase in the older age group. Consumption of yellow wine is concentrated in the adult and older age groups, especially reaching its highest level in the older age group, while consumption of shrimp is relatively evenly distributed.

### 3.3. Dietary Exposure Risks of Rare Earth Elements

In Zhejiang Province, the dietary REE exposure risk (Table 5) was lower than the reference dose (51.30 µg/kg BW/day) in all age groups. Children (aged ≤ 6) had the highest levels of REE exposure (3.71 µg/kg BW/day) compared to other age groups. The concentration of REEs in different foods varied. In all ages, vegetables made the largest contribution to REE exposure, especially the young, tender stem, and leafy vegetables. The exposure risk of fruits and shrimp decreased with age, while the exposure risk of tea showed a trend of first increasing and then decreasing, gradually increasing from children to middle age and decreasing to old age. Overall, the risk of dietary exposure to REEs was low across all scenarios and food types and does not pose a significant threat to population health.

#### Dietary Exposure Risks of Five Rare Earth Elements in Five Food Types

Table 6 presents that the HI for Ce exposure under Scenario 1 exceeded 1 in children aged ≤ 6 years, indicating a potential risk of impaired immune functions in the offsprings. In all other food categories and exposure scenarios, HI values for all age groups remained below 1, suggesting no significant health risk. Among all age groups, children aged ≤ 6 years consistently presented the highest HI values, indicating their heightened vulnerability to exposure to these five rare earth elements. Furthermore, vegetables exhibited the highest HQ values for Y, Ce, Nd, and Sm, whereas fruits displayed the highest HQ values for La.

### 3.4. Daily Safe Consumption for Tea Consumer

Table 7 indicates significant variations in tea consumption and the corresponding amounts required to reach the safe limit across different scenarios, based on a standard body weight of 60 kg for individuals aged 18–59 years. The analysis highlights that across all tea types, the required consumption to reach the safe limit is significantly higher than current consumption. Black tea shows the highest required amount under Scenario 1, at 4298.62 g/day, while Tieguanyin tea requires the least in Scenario 2, at 342.23 g/day. These findings emphasize that the required consumption to reach the safe limit is substantially higher than current consumption levels.

## 4. Discussion

In this study, we investigated REE contamination in food products in Zhejiang Province and evaluated the dietary exposure of different age groups in relation to the food consumption of the population and the concentration of contaminants in food products. REE detection rates in food were notably high, with tea and shrimp exhibiting the highest concentrations among the tested food categories. The dietary exposure of the population was lower than the reference dose, and the exposure of children was slightly higher than that of the other populations.

REE detection rate varied across different food types, with tea showing the highest detection rate (100%), which is consistent with findings from studies in Shanghai [32], Lu’an [53], and Zunyi [54]. The high enrichment of REEs in tea can be attributed to its strong absorption capacity, the use of dry tea in the analysis, and specific processing methods such as roasting and fermentation, which may further concentrate REEs. The REE detection rate in shrimp was 100%, which is consistent with the findings of Shaoxing [55] and Guilin [56] (100% detection of REEs in seafood). A study conducted in Zhuhai found that the REE detection rate in shrimp (100%) was higher than that in fish [57]. The high REE content in shrimp may be attributed to its shallow water habitat, which is more susceptible to contamination [58,59]. Moreover, using REE-containing feed additives in aquaculture may further contribute to REE accumulation in shrimp [60]. The detection rates in vegetables generally agreed with those reported by Wang et al. [32] (83.3% detection of REEs in vegetables), except for aquatic vegetables, which showed lower detection rates. This could be due to the shallow root system of aquatic vegetables, which limits their ability to uptake REE. The detection levels of REEs in fruits were similar to those in vegetables, likely because fruits and vegetables are plant foods, and they have similar REE uptake mechanisms from the soil during growth [61].

In this study, tea contained the highest REE levels. Tea was also found to contain higher levels of REEs (1050 µg/kg) than other foods (grains, vegetables, meat, aquatic products, eggs) in Shanghai. In this study, the concentration of REEs in Tieguanyin tea was significantly higher than the concentration in green and black teas. A survey of REEs in tea in China [62] showed that oolong tea had the highest concentration of REEs (2488.7 µg/kg), followed by black tea (1548.6 µg/kg), with green tea having the lowest (1023.7 µg/kg), which is consistent with our findings. This variation can be primarily attributed to the differences in moisture content. Tea leaves undergo roasting, which reduces their moisture content, leading to a higher concentration of REEs in the dry matter. In contrast, vegetables and fruits have a higher moisture content, which dilutes REE concentrations, resulting in lower levels. In addition, the deep root systems of tea plants enable them to absorb more REEs from the soil [63,64]. In comparison, vegetables typically have shallow root systems [65,66] that limit REE uptake. In aquatic environments, water-grown vegetables have low REE concentrations due to the lower REE levels in water compared to soil and their shallow root system. However, aquatic organisms, such as shrimp may accumulate REEs by ingesting sediment, with calcium and chitin in their shells further enhancing REE retention [58,59]. The REEs in rice wine are relatively low and vary greatly, mainly due to the high water content of wine and the adsorption of rare earth on fermentation lees during processing [67]. Overall, the differences in REE concentrations across food types can be explained by factors such as moisture content, root system structure, processing methods, and species-specific bioaccumulation characteristics.

It is essential to consider the detection rates of specific elements and their natural abundance in the environment to further explore the distribution patterns of REEs. In our study, combined with the chondrite normalization curve and clustering heat map, we discovered that the REE with the highest detection rates in food were Ce, La, and Y (Ce > La > Y), followed by Nd, Sc, Pr, Gd, and Dy, and the detection rates of REEs were low. This pattern aligns with previous findings [68] that reported REE* concentrations in food samples in the order La > Ce > Nd > Y. The higher detection rates of Ce, La, and Y are likely attributed to their natural abundance in the Earth’s crust, where they are present at concentrations of 31–66 µg/g, compared to much lower levels for elements, such as Tm and Lu (0.5–0.8 µg/g) [69]. In the vast majority of foods, the LREE/HREE ratio is >2. The ratios of LREEs/HREEs for tea, vegetables, fruits, shrimp, and yellow wine were 2.7, 3.5, 3.9, 2.1, and 1.4, respectively, suggesting that fruits and vegetables have a high potential for LREE enrichment. The LREE/HREE ratio in Chinese soils ranged from 6.68 to 11.59, with an average of 8.89 [70]. The LREE/HREE ratio in water sediments ranged from 6.93 to 11.97, with an average of 9.57 [71]. The lower LREE/HREE ratio in food compared to soil and sediments suggests a relative enrichment of HREEs in certain food products. This accumulation raises concerns about bioaccumulation and potential toxicity, which may pose risks to food safety and human health.

At the single-element level, Ce was the most abundant REE in all food samples, followed by La, Y, and Nd, which differs from the results reported by Wang et al. [72], who reported Ce to be the most abundant REE in marine wild fish. Y was more abundant than La in cephalopods and Sm was more abundant than La and Nd in milk, eggs, fish, and pork. In the present study, Ce and La had the highest REEs, but Yt exceeded Sm and Nd, which may explain the differences across food species. La, Ce, Nd, and Y have been identified as toxic after oral exposure, affecting several physiological systems. La is associated with learning and memory impairment [73], abnormal calcium metabolism, and reduced bone mineral density [74]. Ce exposure has been linked to placental dysfunction, fetal growth restriction, and anemia [75]. Nd causes DNA damage, abnormal cardiovascular and cerebrovascular development [76], and fetal neural tube defects [77]. Y is associated with bone metabolism disorders, including decreased bone mineral density [78].

Combining the pollutant concentration and consumption data, we discovered that the risk of REE exposure in the five types of food examined in Zhejiang Province was within the safe range. Dietary REEs have also been assessed in other regions of China. Dai [79] found that total dietary concentrations of REEs averaged 64.95 µg/kg ww, and the health risk was considered to be acceptable in a study of marketed food products (including grains, vegetables, meat, dairy products, and aquatic products) from 33 cities in China. Zhao et al. [68] studied representative food samples from the Bayan Ebo mining area in Inner Mongolia, China, and measured an average Estimated Daily Intake of 0.275 µg/kg/day for REEs, which is lower than the recommended daily intake (70 µg/kg/day), again indicating an acceptable risk. However, studies have shown that the influence of REEs is associated with decreased intelligence quotient and memory during neurodevelopment in children [80]. Due to their lower body weight, children have a lower tolerance for pollutants. In this study, the EDI of children (aged ≤6) was higher than that of adults, further indicating a higher risk of REE exposure. In the Shandong [27,30] and Guangdong regions [81], children were at a higher risk of ingesting food with REEs than other populations. Therefore, more attention should be paid to the neurological effects of sustained exposure to low REE levels in children.

In this study, the dietary exposure to five REEs in five food types was assessed based on existing toxicological limits. The limits causing impaired immunity in offspring were as follows: La (0.4277 mg/kg BW/day), Ce (0.086 mg/kg BW/day), Y (0.065 mg/kg BW/day) [45,46,47]. The sub-chronic oral toxicity limits were as follows: Y (29 mg/kg/day) [48]. The genotoxicity threshold for acute exposure was Nd (4.57 mg/kg/day) [49]. The limit causing a significant decline in brain dopamine (DA), 5-hydroxytryptamine (5-HT), and NOS activity were Sm (22.36 mg/kg/day) [50] and the limit of impairment to learning and memory was Sm (2.24 mg/kg/day) [50]. Dietary assessments under different exposure scenarios demonstrated that under extreme conditions (95th percentile concentration and consumption), children aged ≤ 6 years exhibited an HI for Ce beyond 1, suggesting a potential risk of impaired immune function in offspring. However, this reference dose relies on studies in pregnant and lactating mice and might not be directly applicable to young children. For all other age groups, the HI values remained below the safety threshold across all scenarios. Therefore, at the single-element level, the intake of these five rare earth elements through the five assessed food categories does not represent a significant health risk. Among all age groups, children aged ≤ 6 years consistently exhibited the highest HI values, primarily due to their lower body weight, resulting in higher expo-sure per body mass unit. Furthermore, vegetables retained the highest HQ values for Y, Ce, Nd, and Sm, whereas fruits displayed the same for La. This pattern could be attributed to the concentration distributions observed across the five food categories; vegetables generally contained substantially higher Y, Ce, Nd, and Sm levels compared to fruits, while La concentrations were markedly increased in fruits relative to vegetables.

Our study found that tea contains high REE concentrations. Given the widespread tea consumption in Zhejiang Province, we assessed the intake required to reach the safety limit under different scenarios. Our findings showed a significant difference between the current level of tea consumption and the amount required to pose a risk. In Zhejiang Province, residents primarily consume tea in brewed form, which means that the actual intake of REEs is further reduced due to the leaching rate. Even at high levels of contamination and high levels of consumption (for people who consume tea directly), the daily tea intake is still well below the level that could threaten safety. Overall, REEs in tea and other foods were within acceptable limits for all age groups.

This study had some limitations. The analyzed food types were limited, and consumption data (2015–2017) and contaminant concentrations (2018–2019) were collected at different times, potentially affecting the exposure estimates. Moreover, the lack of established maximum allowable limits for individual REEs restricts the accuracy of the risk assessment.

Future research should investigate the long-term health effects of low-level REE exposure, particularly in children [82], and elevated risks in mining-area populations [83]. Assessments of individual REEs, especially HREEs, are needed to address their potential toxicity. Furthermore, comprehensive evaluations of dietary REE intake from food and drinking water remain insufficient, requiring systematic risk assessments [84].

## 5. Conclusions

Tea and shrimp had the highest REE detection rates, with tea exhibiting the highest REE concentration. The detection order of REEs was Ce > La > Y > Nd > Sc > Pr > Gd > Dy, and the LREE/HREE ratio was > 2, but lower than that in the soil and sediment, indicating a potential risk of HREE contamination. Dietary exposure to REEs among Zhejiang residents remained below the health reference values, with children experiencing the highest intake (3.71 µg/kg BW/day) due to their lower body weight. While a potential Ce exposure risk was identified in children ≤ 6 years under extreme conditions, the reference dose was based on studies in pregnant and lactating mice, which might not be directly applicable to this age group. Overall, exposure to all five rare earth elements remained within toxicologically safe limits. The assessment of tea consumption confirmed that REE intake from tea was well below safety thresholds, posing no health risks. Therefore, the findings suggest that REE dietary exposure remains within safe limits under the current dietary structure.

## Figures and Tables

**Table 2:** REE detection in five types of food in Zhejiang Province (%).

Food Category	Detection Rate																		
	REEs	LREE	HREE	La	Ce	Pr	Nd	Sm	Eu	Gd	Tb	Dy	Ho	Er	Tm	Yb	Lu	Sc	Y
Tea																			
Black tea	100	100	100	97	97	97	100	100	100	100	80	100	97	97	89	86	94	91	86
Green tea	100	100	100	100	100	93	100	99	97	100	83	100	93	95	88	89	88	87	94
Tieguanyin tea	100	100	100	100	100	100	100	100	91	100	100	100	100	100	100	100	100	100	91
Vegetable																			
Root vegetables	86	86	69	75	86	51	78	39	16	47	6	37	6	16	2	14	2	53	67
Young stems, leaves, and cauliflowers	93	86	81	75	84	53	66	51	52	37	32	58	40	46	7	43	6	60	67
Fruits and vegetables	83	75	58	67	75	17	60	17	0	8	0	17	0	0	0	0	0	33	50
Fresh bean curd and bean sprouts	100	100	100	50	100	0	0	0	0	0	0	50	0	0	0	0	0	0	100
Aquatic vegetables	47	42	42	37	42	11	39	5	0	11	0	16	0	5	0	0	0	11	32
Fruit																			
Kernels and nuts	81	74	59	59	41	15	26	4	7	7	0	4	0	0	0	0	0	52	19
Berries	100	94	56	81	84	28	14	3	3	19	0	0	0	0	3	0	0	19	31
Tropical and subtropical fruits	100	91	39	70	61	9	5	5	7	14	0	14	0	0	0	0	0	18	23
Other																			
Shrimp and products	100	100	100	100	100	100	100	100	100	100	75	100	90	100	10	100	5	100	100
Yellow wine	84	76	66	14	74	0	60	0	0	0	0	0	0	0	0	0	0	6	62

**Table 3:** Median REE concentrations in five types of food in Zhejiang Province (μg/kg).

Food Category	La *	Ce *	Pr *	Nd *	Sm *	Eu *	Gd ^▲^	Tb ^▲^	Dy ^▲^	Ho ^▲^	Er ^▲^	Tm ^▲^	Yb ^▲^	Lu ^▲^	Sc ^▲^	Y ^▲^	REEs	LREE	HREE	LREE/HREE
Tea																				
Black tea	158	242	26	99	24	7.4	24	3.4	19	4.3	12	2	9.4	2.4	43.1	110	948.8	579	213.3	2.7
Green tea	135.5	184	19	79.2	16.5	5.8	17.7	2.2	14.8	2.7	8	1.2	6.2	1.2	21.6	82.2	777.6	465.8	162.6	2.9
Tieguanyin tea	589	1160	140	537	81.1	18.7	104	16.3	113	24.3	78.8	13.9	103	17.9	132	660	3966.8	3012.8	1421.9	2.1
Vegetable																				
Root vegetables	3.1	4.5	0.7	2	0	0	0	0	0	0	0	0	0	0	1.2	1	30.2	10.5	3	3.5
Young stems, leaves, and cauliflowers	5.3	11.7	0.8	2.3	0.5	0.2	0	0	0.4	0	0	0	0	0	1.8	2.8	81.6	25.3	4.3	5.9
Fruits and vegetables	1.2	2.3	0	0.3	0	0	0	0	0	0	0	0	0	0	0	0.2	6.4	3.5	1	3.5
Fresh bean curd and bean sprouts	0.9	5.4	0	-	0	0	0	0	0.1	0	0	0	0	0	0	1.3	7.6	6.2	1.4	4.4
Aquatic vegetables	0	0	0	0	0	0	0	0	0	0	0	0	0	0	0	0	13.2	0	0	-
Fruit																				
Kernels and nuts	2.9	0	0	0	0	0	0	0	0	0	0	0	0	0	1.3	0	30	6.7	1.7	3.9
Berries	2.8	3.3	0	0	0	0	0	0	0	0	0	0	0	0	0	0	16.6	7.7	0.7	11
Tropical and subtropical fruits	1.5	1.5	0	0	0	0	0	0	0	0	0	0	0	0	0	0	16.7	4.5	0	-
Other																				
Shrimp and products	9.8	9.7	1.1	3.9	0.8	0.2	0.9	0.1	0.6	0.1	0.3	0	0.2	0	3.4	6.1	39.2	26.1	12.2	2.1
Yellow wine	0	1.1	0	0.6	0	0	0	0	0	0	0	0	0	0	0	1.1	3	1.7	1.2	1.4
Others	359	534	62	231	52.4	14.1	56	8.2	42	9.4	26.4	4.1	26	4.2	95.1	284	2051.4	1331.1	554.9	2.4

Note: Elements marked with an asterisk (*) are LREEs; Elements marked with an asterisk (^▲^) are HREEs.

**Table 4:** Consumption of foodstuffs by different age groups of Zhejiang Province residents (g/day).

Food Category	≤6		7–12		13–17		18–59		≥60	
	P50	P95	P50	P95	P50	P95	P50	P95	P50	P95
Tea										
Black tea	0	0	3	3	3	3	3	7.4	2	6.1
Green tea	0	0	1	1	3	5.3	3	10	4	11.8
Tieguanyin tea	0	0	0	0	0	0	3	10	6	6
Vegetable										
Root vegetables	40	120	60	150	71	159.9	90	200	100	200
Young stems, leaves, and cauliflowers	50	130	80	150	100	200	100	212	105	220
Fruits and vegetables	50	131.7	60	150	80	174.4	100	200	100	200
Fresh bean curd and bean sprouts	30	120	60	124.5	70	150	85	180	90	180
Aquatic vegetables	35	101	50	140	77.5	146.5	80	160	82	181.2
Fruit										
Kernels and nuts	100	230	123.5	240	150	250	150	258	150	258
Berries	80	200	100	200	100	200	100	240	105	250
Tropical and subtropical fruits	96	200	100	216.5	115	250	120	250	120	250
Other										
Shrimp and products	45	100	50	147.5	60	153	52	150	50	125
yellow wine	0	0	0	0	0	0	35	500	60.8	621.3

**Table 5:** Dietary exposure risks of rare earth elements (REEs) in five food types across various scenarios (mg/kg BW/day).

Food Category	Scenario 1					Scenario 2				
	≤6	7–12	13–17	18–59	≥60	≤6	7–12	13–17	18–59	≥60
Tea										
Black tea	0	0.14	0.1	0.23	0.2	0	0.23	0.15	0.12	0.08
Green tea	0	0.03	0.13	0.22	0.27	0	0.05	0.10	0.09	0.12
Tieguanyin tea	0	0	0	0.76	0.48	0	0	0	0.3	0.61
vegetable										
Root vegetables	0.43	0.31	0.24	0.27	0.28	0.24	0.2	0.15	0.16	0.18
Young stems, leaves, and cauliflowers	1.43	0.96	0.93	0.88	0.96	0.92	0.83	0.66	0.54	0.58
Fruits and vegetables	0.08	0.05	0.05	0.05	0.05	0.05	0.03	0.03	0.03	0.03
Fresh bean curd and bean sprouts	0.04	0.02	0.02	0.02	0.02	0.02	0.02	0.01	0.01	0.02
Aquatic vegetables	0.13	0.1	0.08	0.08	0.09	0.07	0.06	0.06	0.05	0.05
fruit										
Kernels and nuts	0.73	0.44	0.33	0.31	0.32	0.53	0.37	0.28	0.23	0.24
Berries	0.33	0.19	0.14	0.15	0.16	0.22	0.15	0.1	0.08	0.09
Tropical and subtropical fruits	0.37	0.23	0.20	0.17	0.18	0.30	0.17	0.13	0.11	0.11
Other										
yellow wine	0	0	0	0.05	0.06	0.14	0.08	0.06	0.05	0.05
Shrimp and products	0.18	0.15	0.12	0.1	0.09	0	0	0	0	0.01
*ΣEDI*	3.71	2.66	2.32	3.27	3.17	2.48	2.20	1.73	1.78	2.16
*RfD*	51.30									
*HI*	0.07	0.05	0.05	0.06	0.06	0.05	0.04	0.03	0.03	0.04

Scenario 1: Concentration of rare earth elements in food at 95 percent and consumption of corresponding food at 95 percent. Scenario 2: Concentration of rare earth elements in food at 95 percent and consumption of corresponding food at 50 percent; the risk assessment of tea is calculated by the tea leaching rate.

**Table 6:** Estimated hazard quotients (HQs) of rare earth elements from food consumption across age groups under two exposure scenarios.

Element	Category	Scenario	HQs					*RfDs* *
			≤6	7–12	13–17	18–59	≥60	
Y	Tea	1	0.00	0.00	0.00	0.03	0.03	a
		2	0.00	0.00	0.00	0.00	0.00	
	Vegetable	1	0.34	0.25	0.19	0.16	0.19	
		2	0.19	0.16	0.13	0.09	0.09	
	Fruit	1	0.03	0.03	0.03	0.03	0.03	
		2	0.03	0.03	0.00	0.00	0.00	
	Aquatic	1	0.09	0.06	0.06	0.06	0.03	
		2	0.06	0.03	0.03	0.03	0.03	
	Yellow wine	1	0.00	0.00	0.00	0.03	0.06	
		2	0.00	0.00	0.00	0.00	0.00	
	HI ^▲^	1	0.47	0.34	0.28	0.31	0.34	
		2	0.28	0.22	0.16	0.13	0.13	
La	Tea	1	0.00	0.00	0.00	<0.01	<0.01	a
		2	0.00	0.00	0.00	0.00	0.00	
	Vegetable	1	0.02	0.01	0.01	0.01	0.01	
		2	0.01	0.01	0.01	0.00	0.00	
	Fruit	1	0.18	0.11	0.09	0.08	0.09	
		2	0.13	0.08	0.06	0.05	0.05	
	Aquatic	1	0.02	0.02	0.01	0.01	0.01	
		2	0.01	0.01	0.01	0.00	0.00	
	Yellow wine	1	0.00	0.00	0.00	<0.01	<0.01	
		2	0.00	0.00	0.00	0.00	0.00	
	HI ^▲^	1	0.22	0.14	0.11	0.11	0.12	
		2	0.15	0.10	0.08	0.06	0.06	
Ce	Tea	1	0.00	0.00	0.02	0.02	0.02	a
		2	0.00	0.02	0.00	0.00	0.02	
	Vegetable	1	0.79	0.58	0.42	0.40	0.42	
		2	0.42	0.37	0.30	0.23	0.26	
	Fruit	1	0.12	0.07	0.07	0.05	0.07	
		2	0.09	0.05	0.05	0.02	0.02	
	Aquatic	1	0.12	0.09	0.07	0.07	0.05	
		2	0.09	0.05	0.05	0.02	0.02	
	Yellow wine	1	0.00	0.00	0.00	0.05	0.05	
		2	0.00	0.00	0.00	0.00	0.00	
	HI ^▲^	1	1.02	0.74	0.58	0.58	0.60	
		2	0.60	0.49	0.40	0.28	0.33	
Nd	Tea	1	0.00	0.00	0.00	0.00	0.00	b
		2	0.00	0.00	0.00	0.00	0.00	
	Vegetable	1	<0.01	<0.01	<0.01	<0.01	<0.01	
		2	<0.01	<0.01	<0.01	<0.01	<0.01	
	Fruit	1	0.00	0.00	0.00	0.00	0.00	
		2	0.00	0.00	0.00	0.00	0.00	
	Aquatic	1	<0.01	<0.01	<0.01	<0.01	<0.01	
		2	<0.01	<0.01	<0.01	<0.01	<0.01	
	Yellow wine	1	0.00	0.00	0.00	0.00	0.00	
		2	0.00	0.00	0.00	0.00	0.00	
	HI ^▲^	1	<0.01	<0.01	<0.01	<0.01	<0.01	
		2	<0.01	<0.01	<0.01	<0.01	<0.01	
Sm	Tea	1	0.00	0.00	0.00	0.00	0.00	c
		2	0.00	0.00	0.00	0.00	0.00	
	Vegetable	1	<0.01	<0.01	<0.01	<0.01	<0.01	
		2	<0.01	<0.01	<0.01	<0.01	<0.01	
	Fruit	1	0.00	0.00	0.00	0.00	0.00	
		2	0.00	0.00	0.00	0.00	0.00	
	Aquatic	1	0.00	0.00	0.00	0.00	0.00	
		2	0.00	0.00	0.00	0.00	0.00	
	Yellow wine	1	0.00	0.00	0.00	0.00	0.00	
		2	0.00	0.00	0.00	0.00	0.00	
	HI ^▲^	1	<0.01	<0.01	<0.01	<0.01	<0.01	
		2	<0.01	<0.01	<0.01	<0.01	<0.01	

Scenario 1: Concentration of REEs in food at 95% and consumption of corresponding food at 95%. Scenario 2: Concentration of rare earth elements in food at 95% and consumption of corresponding food at 50%. **^▲^**: HI—Hazard Index, the sum of HQs. *: RfDs based on the existing toxicity limits for animal experiments; the limits were calculated by converting un-certainty factors. a: Limits that cause impaired immunity in offspring. b: Genotoxicity threshold for acute exposure. c: Limits that significantly affect brain dopamine (DA) and 5-hydroxytryptamine (5-HT) levels, as well as total nitric oxide synthase (NOS) activity.

**Table 7:** The consumption of three kinds of tea in different scenarios and daily safe consumption (g/day).

Tea Category	Scenario	Current Consumption	Daily Safe Consumption
Black Tea	1	3	857.20
	2	7.4	4298.62
Green Tea	1	3	1189.67
	2	10	5219.01
Tieguanyin Tea	1	3	342.23
	2	10	682.80

Note: The daily safe consumption of tea was estimated using a reference dose of 51.30 μg/kg BW/day for REEs, based on an adult body weight of 60 kg. After accounting for REE exposure from other food categories under each scenario, the remaining allowable intake was used to back-calculate the maximum amount of tea (g/day) that can be safely consumed for each tea type, based on its REE concentration.

## Data Availability

The original contributions presented in this study are included in the article/Supplementary Materials. Further inquiries can be directed to the corresponding author.

## Supplementary Materials

The following supporting information can be downloaded at: https://www.mdpi.com/article/10.3390/foods14111963/s1, Table S1: Range of REE concentrations in five types of food in Zhejiang Province (g/kg).
