# Supplementary material for: Occurrence and Exposure Assessment of Rare Earth Elements in Zhejiang Province, China
Source: Foods. 2025 May 30;14(11):1963. doi: 10.3390/foods14111963 (PMC12155141; doi:10.3390/foods14111963)
Supplement: Supplementary file 1 [file foods-14-01963-s001.zip › foods-3659273-supplementary.pdf]

Table S1. Range of REEs concentrations in five types of food in Zhejiang Province (μg/kg)

food category	La*	Ce*	Pr*	Nd*	Sm*	Eu*	Gd▲	Tb▲	Dy▲	Ho▲	Er▲	Tm▲	Yb▲	Lu▲	Sc▲	Y▲	REEs	LREE	HREE
**Tea**																			
Black Tea	(0-780)	(0-953)	(0-148)	(4.7-505)	(3.5-90.8)	(1.1-18)	(3.5-102)	(0-37)	(2-73.9)	(0-16.2)	(0-48.7)	(0-8.1)	(0-52.3)	(0-8.7)	(0-170)	(0-510)	(149-3366.1)	(27-2450.5)	(26.8-915.6)
Green Tea	(19.2-2500)	(22-1120)	(0-93.8)	(7.1-376)	(0-474)	(0-41)	(1.2-93.1)	(0-16.1)	(1.1-114)	(0-28)	(0-97)	(0-17.1)	(0-126)	(0-22.1)	(0-98)	(0-865)	(165.6-3041.2)	(103-2594.4)	(30.2-1434.5)
Tieguanyin Tea	(86-903)	(30.7-2760)	(5-642)	(7.7-820)	(6-184)	(0-114)	(18.9-477)	(3.8-54.1)	(26-324)	(7.5-70.8)	(14.5-245)	(4.5-27.3)	(4.4-341)	(7-55.2)	(10-353)	(0-1900)	(314.3-6472.4)	(165.6-4286)	(148.7-3295.8)
**Vegetable**																			
Root vegetables	(0-82.8)	(0-86.6)	(0-8.2)	(0-25.7)	(0-5)	(0-1)	(0-3.2)	(0-0.6)	(0-3.1)	(0-0.6)	(0-1.4)	(0-0.2)	(0-1.1)	(0-0.2)	(0-8.4)	(0-14.7)	(0-220.8)	(0-206.9)	(0-30.4)
Young stems, leaves and cauliflowers	(0-182)	(0-374)	(0-42.3)	(0-162)	(0-31)	(0-6.4)	(0-35.5)	(0-3.6)	(0-16.7)	(0-2.8)	(0-7.2)	(0-0.8)	(0-5.8)	(0-0.6)	(0-42.8)	(0-82.2)	(0-976)	(0-797.7)	(0-178.3)
Fruits and vegetables	(0-9.6)	(0-8.9)	(0-0.9)	(0-2.5)	(0-0.6)	(0-0)	(0-0.3)	(0-0)	(0-0.3)	(0-0)	(0-0)	(0-0)	(0-0)	(0-0)	(0-2.2)	(0-1.7)	(0-20)	(0-16.2)	(0-3.8)
Fresh bean curd and bean sprouts	(0-1.7)	(2.4-8.3)	(0-0)	-	(0-0)	(0-0)	(0-0)	(0-0)	(0-0.2)	(0-0)	(0-0)	(0-0)	(0-0)	(0-0)	(0-0)	(0.8-1.8)	(5-10.3)	(4.1-8.3)	(0.8-2)
Aquatic vegetables	(0-23)	(0-44.7)	(0-5.5)	(0-20.7)	(0-4.5)	(0-0)	(0-3.9)	(0-0)	(0-2.2)	(0-0)	(0-0.8)	(0-0)	(0-0)	(0-0)	(0-9.4)	(0-12.2)	(0-124.7)	(0-98.4)	(0-26.3)
**Fruit**																			
Kernels and nuts	(0-100)	(0-9.9)	(0-2.2)	(0-7)	(0-8)	(0-0.7)	(0-0.8)	(0-0)	(0-0.4)	(0-0)	(0-0)	(0-0)	(0-0)	(0-0)	(0-20)	(0-2.7)	(0-120.3)	(0-100.3)	(0-20)
Berries	(0-200)	(0-13.5)	(0-1.8)	(0-0.7)	(0-0.5)	(0-0.9)	(0-0.6)	(0-0)	(0-0)	(0-0)	(0-0)	(0-0.1)	(0-0)	(0-0)	(0-12)	(0-2.1)	(1.2-200)	(0-200)	(0-12)
Tropical and subtropical fruits	(0-130)	(0-9.8)	(0-1)	(0-1)	(0-0.6)	(0-0.4)	(0-0.8)	(0-0)	(0-0.5)	(0-0)	(0-0)	(0-0)	(0-0)	(0-0)	(0-14)	(0-4.4)	(0.2-130)	(0-130)	(0-18.6)
Shrimp and products	(8.4-13.9)	(6.8-18.9)	(0.9-1.6)	(3.1-6.1)	(0.6-1.2)	(0.1-0.3)	(0.6-1.2)	(0-0.2)	(0.4-0.9)	(0-0.2)	(0.2-0.5)	(0-0.1)	(0.2-0.3)	(0-0.1)	(1.7-6.3)	(4.6-9.1)	(30.8-55.7)	(21.6-40.8)	(8.6-16.4)
Yellow wine	(0-2.4)	(0-4.8)	(0-0)	(0-1.9)	(0-0)	(0-0)	(0-0)	(0-0)	(0-0)	(0-0)	(0-0)	(0-0)	(0-0)	(0-0)	(0-3.2)	(0-2.7)	(0-9.4)	(0-7.2)	(0-4.2)
Others	(36-1010)	(75-2620)	(3.4-238)	(6.6-930)	(8.8-197)	(1.6-52)	(6-164)	(1.4-32)	(13.6-179)	(2.8-35)	(7.1-100)	(0-18)	(0-130)	(1-20)	(4.1-263)	(0-960)	(291.1-6505)	(204.9-5047)	(49.4-1646)

NOTE: Elements marked with an asterisk (*) are LREE；Elements marked with an asterisk (▲) are HREE.
